# Identification of the inhibition mechanism of carbonic anhydrase II by fructooligosaccharides

**DOI:** 10.3389/fmolb.2024.1398603

**Published:** 2024-05-28

**Authors:** Yue Mu, Qingyang Meng, Xinyi Fan, Shuyun Xi, Zhongli Xiong, Yihua Wang, Yanling Huang, Zhen Liu

**Affiliations:** ^1^ School of Chemical Engineering, East China University of Science and Technology, Shanghai, China; ^2^ Shanghai Pechoin Biotechnology Co., Ltd., Shanghai, China; ^3^ Shanghai Zhengxin Biotechnology Co., Ltd., Shanghai, China

**Keywords:** Polygonatum sibiricum, small molecule inhibitor, molecular dynamics simulation, molecular docking, inhibition mechanism

## Abstract

Polygonatum sibiricum (P. sibiricum), recognized as a precious nourishing Chinese traditional medicine, exhibits the pharmacological effect of anti-aging. In this work, we proposed a novel mechanism underlying this effect related to the less studied bioactive compounds fructooligosaccharides in P. sibiricum (PFOS) to identify the inhibition effect of the small glycosyl molecules on the age-related zinc metalloprotease carbonic anhydrase II (CA II). Molecular docking and molecular dynamics simulation were used to investigate the structural and energetic properties of the complex systems consisting of the CA II enzyme and two possible structures of PFOS molecules (PFOS-A and PFOS-B). The binding affinity of PFOS-A (−7.27 ± 1.02 kcal/mol) and PFOS-B (−8.09 ± 1.75 kcal/mol) shows the spontaneity of the binding process and the stability of the combination in the solvent. Based on the residue energy decomposition and nonbonded interactions analysis, the C-, D- and G-sheet fragments of the CA II were found to be crucial in binding process. Van der Waals interactions form on the hydrophobic surface of CAII mainly with 131PHE and 135VAL, while hydrogen bonds form on the hydrophilic surface mainly with 67ASN and 92GLN. The binding of PFOS results in the blocking of the zinc ions pocket and then inhibiting its catalytic activity, the stability of which has been further demonstrated by free energy landscape. These findings provide evidence of the effective inhibition of PFOS to CA II enzyme, which leads to a novel direction for exploring the mechanism of traditional Chinese medicine focused on small molecule fructooligosaccharides.

## Graphical Abstract

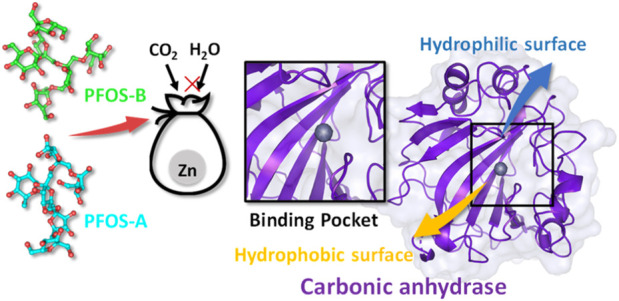

## 1 Introduction

Polygonatum sibiricum (P. sibiricum), known as the “GOLD” in traditional Chinese medicine, is a medicinal and food homologous plant belonging to the Liliaceae family. (Wang et al., 2019). It has been employed as a constituent of traditional medicinal formulations with multifaceted pharmacological properties for over 2000 years (An et al., 2006; Han et al., 2020; Shen et al., 2022). In recent years, considerable research efforts have been focused on investigating the potential health benefits of P. sibiricum (Wang et al., 2019; Li et al., 2021; Liu et al., 2022). Various bioactive compounds such as polysaccharides, flavonoids, and steroidal saponins have been extracted.

The anti-aging function of P. sibiricum has received considerable attention among its various pharmacological applications. Multiple mechanisms of this function have been proposed, primarily through its antioxidative properties, protection of cellular components, and improvement in metabolic functions (Cui et al., 2018). However, related research primarily revolves around the polysaccharides. Scant attention was given to other biologically active components such as oligosaccharides, which also possess potential pharmacological properties contributing to anti-aging function of P. sibiricum. A comprehensive understanding of the intricate molecular mechanism underlying this effect remains limited (Zhao et al., 2018; Zheng, 2020; Wang et al., 2022).

**FIGURE 1:**
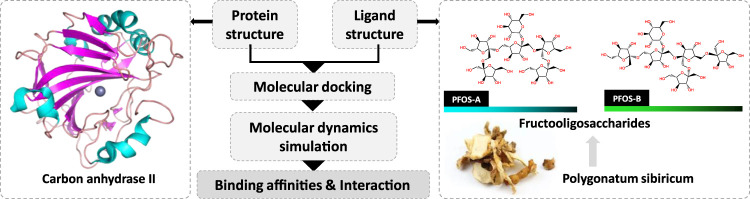
Investigation of the inhibition effect of P. sibiricum Fructooligosaccharides on carbon anhydrase II based on molecular docking and molecular dynamics simulation.

Notable, the significant presence of fructooligosaccharides in medicinal plants and their crucial role in the treatment of various diseases has been proposed in recent research (Chen et al., 2015). The bioactivity of fructooligosaccharides resulting from the hydrolysis of these polysaccharides in P. sibiricum has also been highlighted (Liu et al., 2004; He et al., 2021). This particular component may have the potential to protect the cellular components such as endothelial cells and mitochondria. Given their prominent role and the preliminary evidence supporting their unique contributions to the pharmacological profile of P. sibiricum, it was crucial to isolate and analyze these compounds specifically to clarify their role in anti-aging. The two possible structures of fructooligosaccharides in P. sibiricum (PFOS) was identified in previous research (Figure 1).

Carbonic anhydrase II (CA II) emerges as a possible target of PFOS. It shows a distribution across diverse human organs and tissues. As the first zinc metalloprotease identified in 1933, (Meldrum and Roughton, 1933), CA II represents a pivotal isoform within the spectrum of human carbonic anhydrase (CAs, CA I-CA XIV). Its significance lies in its indispensable involvement in one of the most fundamental reactions in human existence: the conversion of CO_2_ and H_2_O into bicarbonate and protons (Imtaiyaz Hassan et al., 2013; Nocentini et al., 2019; Occhipinti and Boron, 2019). Given the pervasive nature of the reactions, CA II influence a wide range of metabolic processes, including the facilitation of transmembrane CO_2_ and O_2_ transport during the respiration, the mediation of transepithelial fluid and acid-base exchange and the maintenance of the whole-body acid-base equilibrium (Alterio et al., 2012; Supuran, 2021; Angeli et al., 2023).

Actually, the inhibition of the CA II has attracted considerable attention (Nocentini et al., 2019). Based on the identification of the structure of the CA II, physiological functions, and pathological roles, it has been selected as a drug target for the treatment of diverse diseases and impairments, including glaucoma, tumorigenesis, obesity calcification (Lie-Injo et al., 1970; Supuran, 2008). Extensive efforts have been devoted to the design of the CA II inhibitors to treat these diseases and impairments by inhibiting its abnormal expression or reducing its activity (Nocentini et al., 2004; Scozzafava et al., 2006; Carta and Supuran, 2013; Supuran et al., 2015; Occhipinti and Boron, 2019; Ghorai et al., 2020).

Currently, the most active classes of the CA II inhibitors are sulfonamides and their bioisosters, sulfamides, and sulfamates (Supuran et al., 2004; Husain and Madhesia, 2012). These inhibitors play an active role by binding with the catalytic zinc ion in the CA II or blocking the reaction pocket. However, the usage of them is still limited due to the safety concerns arising from the weak selectivity towards the CA II compared to other CA isozymes. Therefore, the search for novel compounds for the CA II inhibition has been motivated by this obstacle (Supuran, 2011; Poulsen and Davis, 2014; Carreyre et al., 2017; Supuran, 2020; Supuran, 2022a; Supuran, 2022b), as these compounds exhibit a favorable profile with lower toxicity and reduced side effects compared to synthetic small molecule inhibitors.

Intriguingly, recent scientific findings have pinpointed a correlation between age-related physiological impairments and the CA II quantity within mitochondria of specific cell types, (Pollard et al., 2016; Feng et al., 2018), which signifies an increase of the CA II activity concurrent with aging. Consequently, the novel compound PFOS may have the potential to inhibit the CA II activity, providing a plausible explanation for the observed anti-aging function of P. sibiricum.

This study aims to comprehensively elucidate the intricate mechanisms underlying the anti-aging properties of P. sibiricum, especially with respect to under-explored bioactive compounds fructooligosaccharides, and to highlight the necessity to identify novel non-toxic inhibitors for the CA II. We studied the inhibition mechanism of PFOS on the CA II for three main objectives: 1) assessment of the binding affinities; 2) comprehensive investigation of the protein-ligand interactions; 3) analysis of the intricate dynamics characteristics through molecular docking and molecular dynamics (MD) simulations.

## 2 Materials and methods

### 2.1 Molecular docking

Molecular docking is a powerful method for high-throughput virtual screening of small-molecule inhibitors and predicting the ligand-protein interactions in computer-assisted drug design. It consists of three main types: rigid docking, semi-flexible docking, and flexible docking, which are categorized in ascending order of protein flexibility. Semi-flexible docking was chosen for its balance between computational efficiency and accuracy, allowing modifications in the conformations of ligands and residues within a defined area.

The crystal structure of the CA II (PDB: 12CA) was sourced from the RCSB library and does not include post-translational modifications (Berman et al., 2000). Both PFOS-A and PFOS-B were fully optimized utilizing the Gaussian09 software (Frisch MJT et al., 2009) at the density functional theory level, employing the B3LYP function with the London-dispersion correction by Grimme and the def2-SVP basis set (Schäfer et al., 1994; Grimme et al., 2011).

Autodock Vina was utilized for the docking of PFOS with CA II (Trott and Olson, 2010). A 10 × 10 × 10 nm box, set up using default parameters, encompassed almost the entire active surface of CA II, centered on the typical binding pocket identified in previous research. This pocket, delineated by hydrophobic residues (Ile91, Val121, Phe131, Leu141, Val143, and Leu198) and hydrophilic residues (Asn62, His64, Asn67, and Gln92), is crucial for rapid catalytic conversion of CO_2_ to bicarbonate. The energy range was set to 5.0 kcal/mol, and the exhaustiveness parameter was configured as 22 (Agarwal and Smith, 2023). The optimal pose with the best scores within multiple iterations of the docking process was selected as the initial structure for the subsequent MD simulation, according to the affinity assessment through scoring function and binding mode analysis (Tanchuk et al., 2016).

The frontier molecular orbital analysis was conducted to assess the docking reactivity of these molecules due to the significance of HOMO and LUMO (E_HOMO_ and E_LUMO_) energy gaps in chemical reactivity. Various physical and chemical parameters, including chemical hardness (η), softness (s), and electrophilicity index (w), were calculated and analyzed.

### 2.2 Molecular dynamics simulation

Based on the initial structures obtained from the molecular docking results, MD simulations were conducted using the GROMACS 2016 software package, (Abraham et al., 2015), and the proteins and small molecules were parameterized by the all-atom CHARMM36 and CHARMM general force field (CGenFF), respectively.

The complex was put in a 10 × 10 × 10 nm periodic cubic box and then filled with the TIP3P water molecules. (Mark and Nilsson, 2001). Then, the chloride ions were added to neutralize the system charge. During the energy minimization process, the unreasonable interactions and atomic collisions were removed using the steepest decent and conjugated gradient methods. Next, the temperature of each system was incrementally increased from 0 K to 300 K during the 1 ns simulation using an NVT ensemble. The temperature of the protein-ligand and solvent-ion groups was controlled separately using a Berendsen thermostat to obtain more reliable results. After that, a Parrinello–Rahman barostat was used to maintain the pressure of the systems at 0.1 MPa in NPT equilibration for 1 ns. Finally, MD simulations were conducted for 100 ns, and the last 10 ns trajectory was taken every 10 ps for subsequent analysis.

During the simulation, the length of the hydrogen bonds was constrained by LINear Constraint Solver algorithm. The cut-off for short-range nonbonded interactions was set at 1.0 nm, while for long-range electrostatic interactions, the Particle Mesh Ewald algorithm was employed without an explicit cut-off. The determination of cut-offs was guided by empirical practices widely adopted in previous studies (Essmann et al., 1995; Kabedev et al., 2023; Sun et al., 2023). Newton’s classical equations of motion were integrated using the Verlet leapfrog algorithm. (Hockney et al., 1974). The stability of the systems was checked by the root mean square deviation (RMSD) of the protein backbone.

### 2.3 Binding free energy

To further assess the interaction between PFOS and CAII, the binding energy was calculated and partitioned into different parts according to the type of interaction by the MM-PBSA method implemented in the gmx_MMPBSA software package (Miller et al., 2012; Suri and Naik, 2015). It should be noted that the binding free energies calculated by the MM-PBSA method is system-dependent and sensitive to the chosen parameters, which is somewhat less accurate compared to the free energy perturbation or thermodynamic integration (Breznik et al., 2023). However, the computationally efficient MM-PBSA method has proven to be reliable in the field of computational drug design and evaluation (Genheden and Ryde, 2015; Wen et al., 2019).

This calculation was conducted using a set of snapshots extracted from the final 10 ns trajectory of the MD simulations, sampled at 10 ps intervals. The binding free energy calculations in the MM-PBSA method can be concisely represented by the following equations:

$$\Delta G=G_{complex}-\left(G_{receptor}+G_{ligand}\right)=\Delta H-T\Delta S$$ (1)

$$\Delta G=\Delta E_{gas}+\Delta G_{sol}-T\Delta S$$ (2)

where,

$$\Delta E_{gas}=\Delta E_{binded}+\Delta E_{nonbinded}$$ (3)

$$\Delta E_{binded}=\Delta E_{bind}+\Delta E_{angle}+\Delta E_{dihedral}$$ (4)

$$\Delta E_{nonbinded}=\Delta E_{ele}+\Delta E_{vdw}$$ (5)

and,

$$\Delta G_{sol}=\Delta G_{polar}+\Delta G_{nonpolar}=\Delta G_{PB}+\Delta G_{SA}$$ (6)

where,

$$\Delta G_{SA}=NP_{TENSION}\times \Delta SASA+NP_{OFFSET}=\gamma \cdot \Delta SASA$$ (7)

Within these equations, G_complex_, G_receptor_, and G_ligand_ are the free energy of the complex system, CA II and small molecule inhibitors, respectively, collectively contributing to the binding free energy (∆G). This free energy can be further partitioned into the sum of the binding enthalpy (∆H) and the conformational entropy change upon inhibitor binding (-T∆S), as depicted in Eq. 1.

The enthalpic term, denoted as ∆H, can be formulated as the sum of ∆E_gas_ and ∆G_sol_, as elaborated in Eq. 2. ∆E_gas_ accounts for the energy changes computed through molecular mechanics in the gas phase, while ∆G_sol_ pertains to solvent-related energy contributions.

In Eqs 3–5, the term ∆E_gas_ is divided into two separate contributions: ∆E_binded_ and ∆E_nonbinded_. ∆E_binded_ encompasses energy contributions arising from binding, angle, and dihedral alterations, while ∆E_nonbinded_ can be further partitioned into the ∆E_ele_ and the ∆E_vdw_.

The computation of the solvation-free energy involves two components: ∆G_polar_, assessed via the Poisson–Boltzmann approach, and the ∆G_nonpolar_, estimated through a SASA-only model (Eq. 6), assuming proportionality to the solvent accessible surface (∆SASA) outlined in Eq. 7.

Notably, the entropy change (∆S) effect is ignored due to computational constraints and the substantial uncertainty it introduces into the results, stemming from considerable errors during the calculation process. Several studies have shown that the MM-PBSA method can give high calculation accuracy without accounting for ∆S (Qin et al., 2010; Hou et al., 2011; Guo et al., 2021).

### 2.4 Simulation analysis

The trajectory obtained from the MD simulation was analyzed using auxiliary tools provided within the GROMACS 2016 package. The interaction between small molecules and CA II receptors is analyzed by Protein-Ligand Interaction Profiler (PLIP). Furthermore, the occupancies of the intermolecular hydrogen bonds within the PFOS systems were calculated using the Visual Molecular Dynamics (VMD) 1.9.3 software. With the focus on direct interaction between PFOS and CAII, the trajectory used in hydrogen binding analysis have been pre-processed to remove the waters. Hydrogen bonds were identified based on established criteria: an acceptor-hydrogen-donor angle exceeding 135° and an acceptor-hydrogen atom distance less than 3.5 Å.

In addition, a principal component analysis was conducted to delineate the dominant motion of the CA II and the correlated motions among the atoms extracted from the MD trajectories within the PFOSs system (Ma et al., 2017; John et al., 2020). Notably, an intermolecular motion was excluded by eliminating protein translation and rotation via the auxiliary tools available in the GROMACS suite. The eigenvectors, which signify the directions of the atomic motions, along with their corresponding eigenvalues that indicated their magnitudes, were calculated by evaluating the covariance matrix of the α-carbon atoms of the CA II protein. This process aimed to elucidate the impact of ligands on the conformational distribution of the protein (Mu et al., 2005; Papaleo et al., 2009). Subsequently, the first two principal components (PC1 and PC2) were utilized as reaction coordinates to construct the free energy landscape, and the free energy values were calculated with Eq. 8.

$$G=-k_{B}T\times \ln P$$ (8)

Where k_B_ represents Boltzmann constant, T stands for the simulation temperature, and P is the probability of the conformational distribution.

## 3 Results and discussion

### 3.1 Docking of CA II and PFOS

**FIGURE 2:**
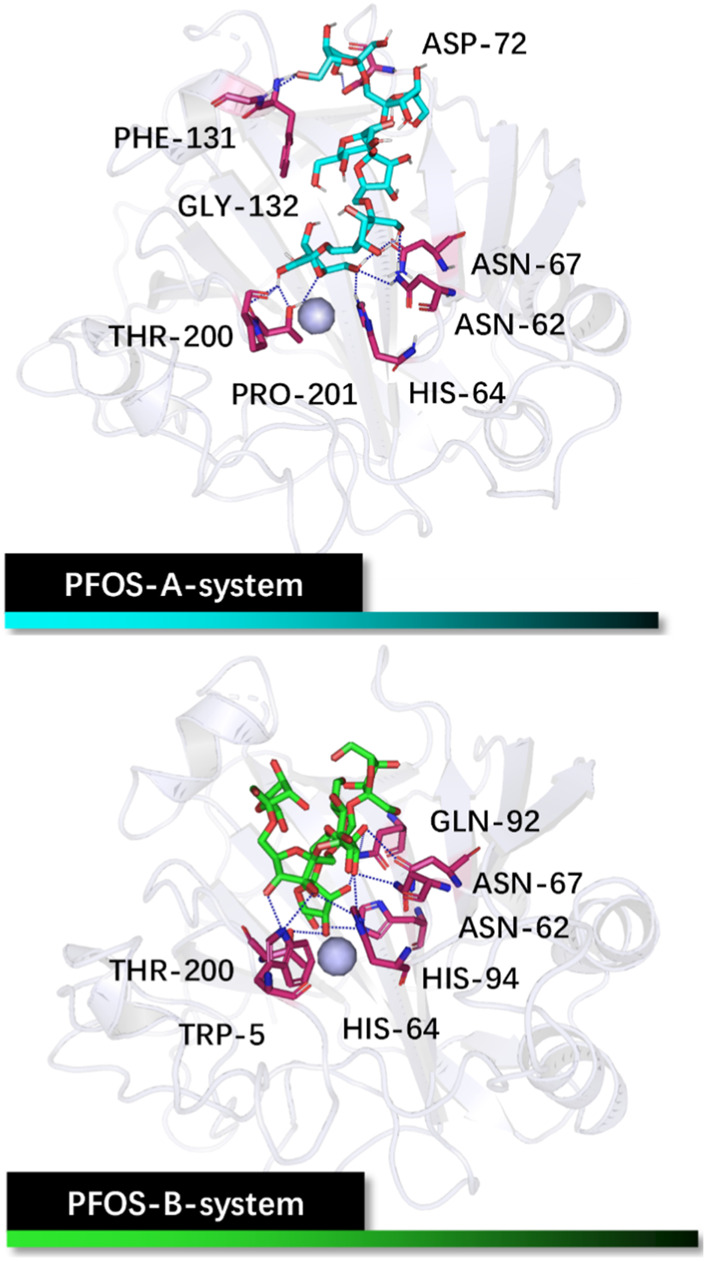
Initial structures of the PFOS-A and PFOS-B bound to CA II. The skeleton of the PFOS-A is in blue, PFOS-B is in green, and the bound residues are in purple.

The initial binding structures of CA II with each PFOS compound were obtained through molecular docking. Firstly, to validate the reliability of the docking method, redocking experiments were performed on the complex of CA II with N-[(4-methylphenyl)methyl]-thiophene-2,5-disulfonamid (PDB: 1BNA, inhibitor: AL5) (Boriack-Sjodin et al., 1998). The results demonstrate that the conformation of the complex obtained by docking method matches with the native crystal structure located in the active pocket of the CA II, as shown in Supplementary Figure S1, with an RMSD of 0.78 Å. Furthermore, the interactions of the docked pose, assessed using the PLIP program, (Adasme et al., 2021), are consistent with the research conducted by Sjodin, (Boriack-Sjodin et al., 1998), further validating the accuracy of the docking method. The correlative analysis is shown in Supplementary Figure S2. Having validated the docking method, the two PFOS molecules were then docked to the CA II using the same docking method (Figure 2). The conformations with the best score for both structures are in the pocket adjacent to the zinc ions.

The docking results reveal that both PFOS structures effectively bind to the active site on CA II as classical inhibitor AL5 used for docking verification. The chemical softness of the small molecules underscores their binding reactivity, with values of 7.58 for PFOS-A and 7.96 for PFOS-B, since a higher value of chemical softness typically correlates with increased molecular reactivity. The additional details on other chemical reactivity parameters are provided in Supplementary Table S1.

Analysis based on PLIP reveals that PFOS-A predominantly binds to residues ASN-62, HIS-64, ASN-67, ASP-72, PHE-131, GLY-132, THR-200, and PRO-201, while PFOS-B exhibits binding interactions with TRP-5, ASN-62, HIS-64, ASN-67, GLN-92, HIS-94 and THR-200. These residues are primarily located on the hydrophilic surface of the protein, attributed to the presence of multiple hydroxyl groups in these glycosyl molecules. However, the neglect of the structural flexibility inherent in the majority of protein structures in the docking method is a formidable obstacle to accurate binding analysis. Nevertheless, the docking conformations can still serve as valuable initial input structures for subsequent MD simulations and as a reference for the binding analysis. The off-target effects of PFOS have also been preliminarily assessed by the molecular docking method (Supplementary Figure S3). It should be noted that the activity of the non-target protein CA I may be slightly affected.

### 3.2 RMSD

**FIGURE 3:**
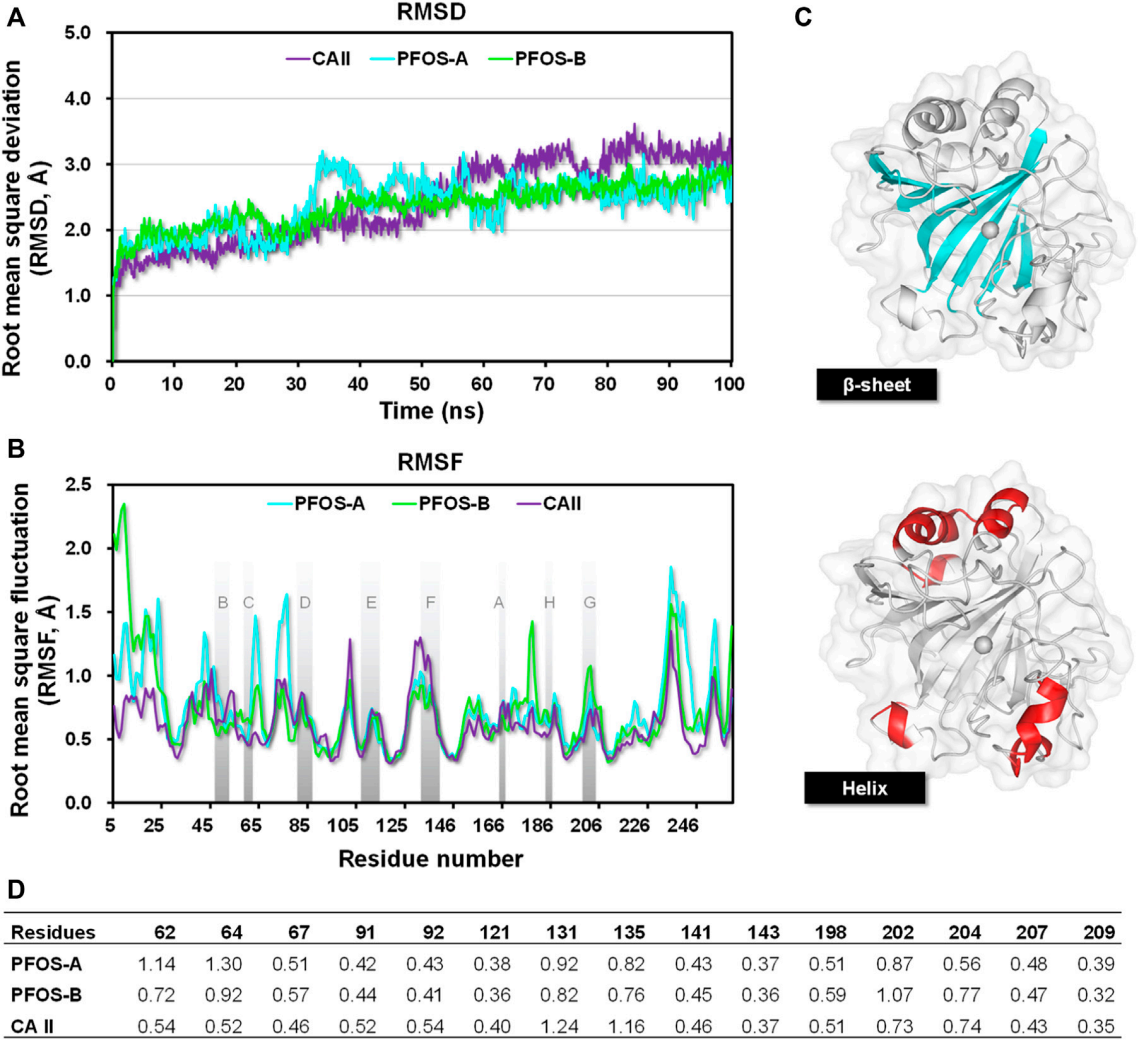
**(A)** The RMSDs of residues reside within 10 Å of the ligand in the CA II, PFOS-A, and PFOS-B systems. **(B)** The RMSF plots of all the systems during 100 ns MD simulation and the β-sheet region are named as reported by Eriksson (Eriksson et al., 1988); **(C)** The secondary structure of the CA II. The β-sheet region is shown in blue, and the helix region is shown in red. **(D)** RMSF of residues located in the active site of CA II.

With the initial structures obtained by molecular docking, 100 ns MD simulations of the systems were performed to get a more detailed and accurate understanding of their behavior. The RMSD was calculated for the resides within 10 Å of the ligand to assess the structural stability throughout the 100 ns simulations. As illustrated in Figure 3A, both PFOS complexes reached equilibrium after 50 ns, exhibiting the RMSD values of less than 2.8 Å for the majority of the equilibration stage, which are more stable than the CA II without inhibitor (averaging 3.0 Å in the last 50 ns). This observation suggests that the presence of the PFOS small molecules stabilized the core backbone of the CA II. Notably, during the equilibration period, the PFOS-B system demonstrated slightly higher stability with lower RMSD values than the PFOS-A system, while the opposite trend was observed during the early stage. This discrepancy may be attributed to the disruption or instability of the hydrogen bonds present in the initial docking structure of the PFOS-A system. Nevertheless, since all three systems reached equilibrium, further analysis can be conducted based on the obtained MD simulation trajectory.

### 3.3 RMSF

The Root mean square fluctuation (RMSF) was further calculated to measure the per-residue flexibility of the CA II, PFOS-A, and PFOS-B complex systems. As illustrated in Figure 3B, the closely overlapping profiles of PFOS complexes and CA II suggest that the inhibitor has little effect on the regional flexibility of the protein. The stable regions mainly occurred at the β-sheets with the RMSF less than 0.8 Å, while the helix domain showed higher RMSF values up to 2.5 Å.

The secondary structure of CA II is shown in Figure 3C, which consists of 10 stranded twisted β-sheet and 7 helices and the zinc ions presented in the center bottom of the protein chain surrounded by the β-sheet regions. According to the secondary structure, all helix regions with high RMSF values were far away from the binding pocket and had little effect on the binding process. On the contrary, the β-sheet, especially the C-sheet, D-sheet, and G-sheet segments, showed relatively lower RMSF values in complex with PFOS as inhibitors compared to the CA II system. These segments are identified as the key sites for the combination of PFOS. In addition, systems with PFOS-B as the inhibitor are found to have higher residue stability than PFOS-A, which is in accordance with the previous RMSD analysis.

Further RMSF analysis focused on residues within the active site of CA II (Figure 3D). Upon binding with PFOS, the flexibility of these residues generally decreased, particularly for residues 131PHE and 135VAL. These two residues have also been identified as key contributors to the protein-ligand interaction in subsequent energy analysis to accommodate the hydroxyl group of PFOS.

### 3.4 Binding free energy

It has been shown that the PFOS effectively stabilize the CA II protein via binding with some key residues situated within the β-sheet region. However, the lack of a zinc-binding group distinguishes them from most existing CA II-targeting inhibitors. It is difficult for PFOS structures to bind directly to the catalytic zinc ion, which plays a critical role in the catalytic function of the enzyme. Hence, it is crucial to validate the exact binding affinities of these two small molecules with CA II, wherein the MM-PBSA method was employed. This calculation relied on the snapshots extracted from the final 10 ns of the equilibrium stage in the MD simulation of each respective system. The same methodology was also applied to the experimentally tested inhibitor AL5, employed in the docking validation, serving as the benchmark (Table 1).

**TABLE 1:** Binding energies in PFOS-A, PFOS-B, and CA II systems by MM-PBSA method (kcal/mol).

Contribution	PFOS-A	PFOS-B	AL5
∆E_ele_ ^a^	−13.15 ± 1.78	−31.92 ± 2.64	−8.64 ± 1.82
∆E_vdw_ ^b^	−26.53 ± 1.03	−35.49 ± 1.07	−23.94 ± 0.99
∆E_PB_ ^c^	36.23 ± 1.55	64.58 ± 3.25	18.94 ± 1.00
∆E_SA_ ^d^	−3.83 ± 0.17	−5.26 ± 0.15	−2.92 ± 0.06
∆E_polar, total_ ^e^	23.08 ± 3.33	32.66 ± 5.89	10.32 ± 2.82
∆E_nonpolar, total_ ^f^	−30.36 ± 1.20	−40.75 ± 1.22	−26.86 ± 1.05
ΔG^g^	−7.27 ± 1.02	−8.09 ± 1.75	−16.56 ± 0.73

^a^ Electrostatic energy.

^b^ Van der Waals energy.

^c^ Polar solvation free energy.

^d^ Non-polar solvation free energy.

^e^ Total polar binding free energy (consisting of electrostatic energy and polar solvation free energy).

^f^ Total non-polar binding free energy (consisting of Van der Waals energy and the nonpolar solvation free energy).

^g^ Binding free energy.

The two PFOS structures show comparable binding affinity to the CA II protein (Table 1). The PFOS-B (−8.09 kcal/mol) shows a slightly lower ΔG compared to PFOS-A (−7.27 kcal/mol), a trend consistent with the RMSD and RMSF (Figure 3) results. From a thermodynamic perspective, these negative values corroborate the spontaneity of the binding process and the stability of the combination in the solvent. This level of binding stability aligns with findings in similar research, where a small glycosyl molecule interacts with the human sweet taste receptor, exhibiting a ΔG of −8.53 ± 1.78 kcal/mol, as validated through MD simulations and experimental data. (Mahalapbutr et al., 2020). However, the biological activity of PFOS-A and PFOS-B is relatively lower when compared to typical CA II inhibitors exemplified by the benchmark compound AL5. This difference in activity can be attributed to the absence of the zinc-binding group in the PFOS structure, which can form much more favorable electronic interactions to maintain the combination of the complex.

To further investigate the potential inhibition mechanism of PFOS on CA II, common features of the two structures are first identified by energy decomposition analysis. The total binding energy can be decomposed into different components including electrostatic energy, van der Waals energy, polar solvation-free energy, and non-polar solvation energy. Thus, relative importance of each interaction within the binding process can be assessed.

The dominant propellant for binding for both the PFOS-A and PFOS-B systems is the total nonpolar binding energy, which can be further separated into van der Waals energy and non-polar solvation-free energy (Table 1). Both these components contribute positively to the binding process. In addition, negative electrostatic energy was observed in all systems, with significantly lower values in both PFOS systems compared to AL5. This discrepancy can be attributed to the abundant hydroxyl groups in PFOS-A and PFOS-B (Figure 1), which exhibit substantial potential for establishing hydrogen bonding interactions. However, a significant unfavorable influence was detected concerning the total polar free energy, primarily attributed to the desolvation process, chiefly driven by the considerable polarity exhibited by the PFOS molecules. The formation of intramolecular hydrogen bonds by PFOS molecules may also impose limitations on their interactions with CA II molecules. The slightly weaker binding free energy of PFOS-A observed during dynamic simulations will be explained by following energy decomposition.

### 3.5 Per-residue energy decomposition

**FIGURE 4:**
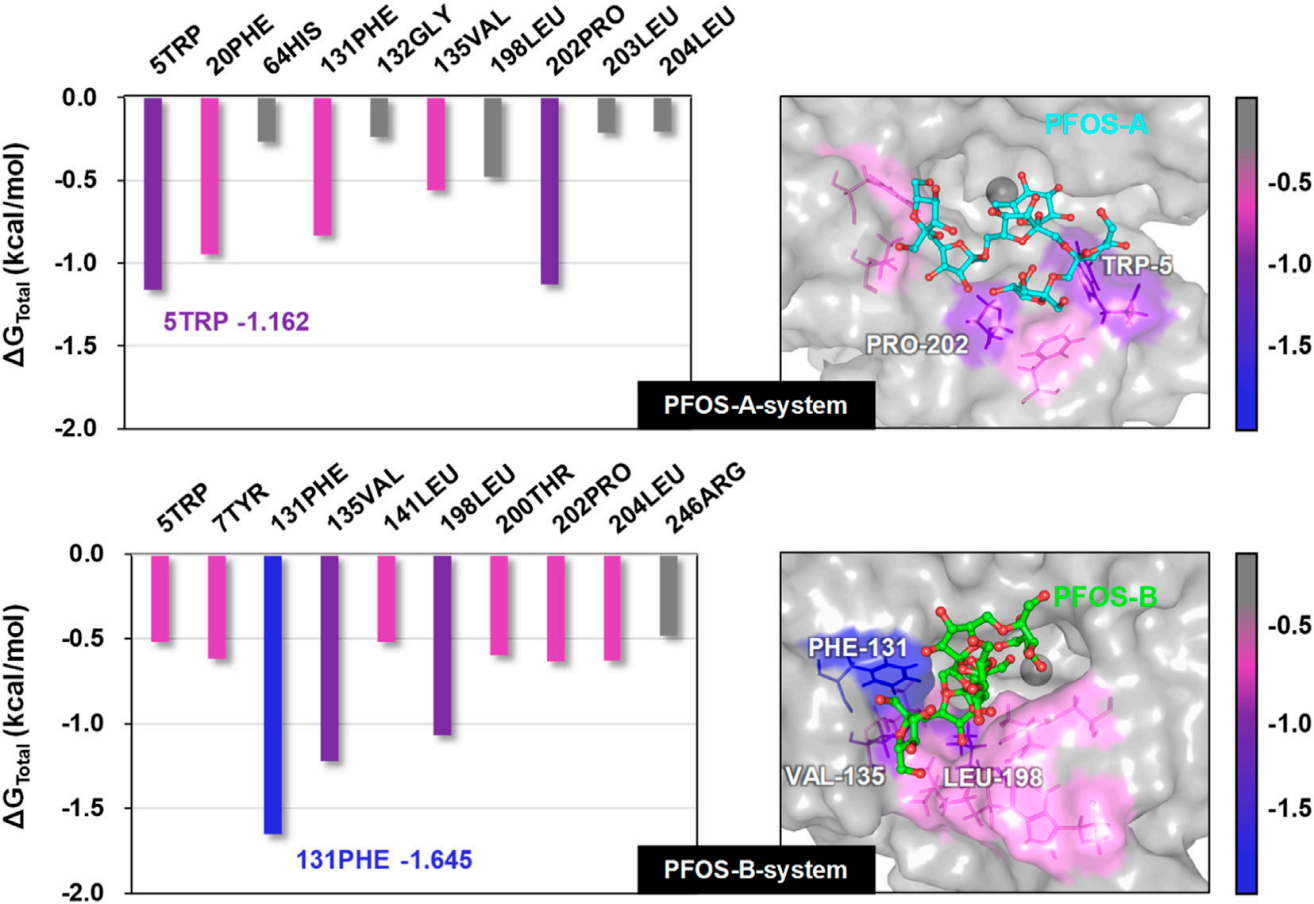
Total binding free energy of PFOS-A and PFOS-B in complex with CA II protein. Representative 3D structures illustrating the binding region of the small molecules on the CA II protein were taken from the last 10 ns MD snapshot with the lowest energy and color-coded according to their binding free energy values.

In order to elucidate the detailed interaction between PFOS molecules and the CA II protein, the identification of key residues critical for the binding process was pursued through energy decomposition analysis. The analysis encompassed residues within a 10 Å radius of the PFOS molecules. The ten residues exhibiting the most substantial contributions to the binding energy were ranked and presented in Figure 4. Key residues identified by contributions exceeding the threshold of −0.5 kcal/mol were summarized in Table 2 and Table 3. The detailed energy decomposition result is shown in Supplementary Tables S2, S3.

**TABLE 2:** Key residues energy decomposition in PFOS-A system (kcal/mol).

Residue	∆E_vdw_	∆E_ele_	∆E_PB_	ΔG
5TRP	−2.564	−1.944	3.346	−1.162
202PRO	−1.312	−0.585	0.768	−1.130
20PHE	−1.063	−0.205	0.319	−0.948
131PHE	−1.404	−0.005	0.577	−0.832
135VAL	−0.507	−0.009	−0.047	−0.563

**TABLE 3:** Key residues energy decomposition in PFOS-B system (kcal/mol).

Residue	∆E_vdw_	∆E_ele_	∆E_PB_	ΔG
131PHE	−2.302	−0.199	0.856	−1.645
135VAL	−1.217	0.143	−0.140	−1.215
198LEU	−1.106	−0.341	0.386	−1.061
202PRO	−0.710	−0.100	0.181	−0.629
204LEU	−0.675	0.099	−0.047	−0.623
7TYR	−2.019	−0.625	2.034	−0.609
200THR	−1.320	−0.382	1.112	−0.591
141LEU	−0.648	−0.085	0.220	−0.512
5TRP	−0.589	−0.459	0.538	−0.510

PFOS-A structure interacted with 5 key residues: TRP-5, PRO-202, PHE-20, PHE-131, and VAL-135 (Figure 4). Meanwhile, the PFOS-B structure interacted with 9 pivotal residues: PHE-131, VAL-135, LEU-198, PRO-202, LEU-204, TYR-7, THR-200, LEU-141, and TRP-5, which is nearly twice the key residues that participated in the combination of PFOS-A system. Based on the analysis of the docking conformation of the two systems, the reason for the fewer key residues in PFOS-A may be attributed to its stick line backbone structure in the combination process, which makes it less possible to access the residues located at the entrance of the docking pocket to form van der Waals interaction with them. The fusiform structure of PFOS-B makes it more suitable to block the docking pocket with more accessible residues. In addition, the van der Waals energy contribution of the key residues is significantly larger compared to the electrostatic energy, which is consistent with the previous analysis in Table 1. The TRP-5 in the PFOS-A system and the PHE-131 in the PFOS-B system exhibited the most favorable ΔG_total_ values. For both PFOS structures, residues PHE-131, VAL-13 were found to play a significant role in the van der Waals interactions at the hydrophobic side of the binding pocket entrance of the CA II protein. These findings are consistent with the prior studies suggesting the importance of these residues in the binding of the hydrophobic tail in Zinc binding group inhibitors. (Alterio et al., 2012).

### 3.6 Binding mode analysis

The electrostatic interaction also plays an important role in inducing the complexation of PFOS molecules and CA II, in addition to van der Waals and the non-polar solvation effect. Further characterization of this kind of interaction anchoring the PFOS small molecules into the binding pocket was conducted. The occupancy of the direct intermolecular hydrogen bonds was statistically analyzed over the last ten MD simulations using the PLIP and Visual Molecular Dynamics (VMD) program. (Humphrey et al., 1996). The percentage occupancy of hydrogen bonds in the PFOS-A and PFOS-B systems are presented in Supplementary Tables S4, S5. The stable hydrogen bonding has been summarized according to the standard for stable hydrogen bonding, that the hydrogen bonding donor-acceptor interaction occupancy is greater than 10% (Table 4; Table 5). As expected, both PFOS systems showed the formation of hydrogen bonds with polar residues within the C-sheet, D-sheet and G-sheet of the CA II protein. However, it can be seen that the number and stability of these hydrogen bond formations are different for the two systems.

**TABLE 4:** Main hydrogen bond occupancies in PFOS-A system.

Donor	Hydrogen	Acceptor	Occupancy%
5TRP@NE1	5TRP@HE1	PFOS-A@O72	39.6
5TRP@NE1	5TRP@HE1	PFOS-A@O70	35.6
67ASN@ND2	67ASN@HD21	PFOS-A@O4	17.8

**TABLE 5:** Main hydrogen bond occupancies in PFOS-B system.

Donor	Hydrogen	Acceptor	Occupancy%
PFOS-B@O18	PFOS-B@H11	201PRO@O	23.8
PFOS-B@O19	PFOS-B@H12	201PRO@O	23.8
PFOS-B@O31	PFOS-B@H20	92GLN@OE1	17.8
PFOS-B@O24	PFOS-B@H15	136GLN@OE1	16.8
PFOS-B@O29	PFOS-B@H18	92GLN@OE1	14.9
62ASN@ND2	62ASN@HD21	PFOS-B@O7	10.9

**FIGURE 5:**
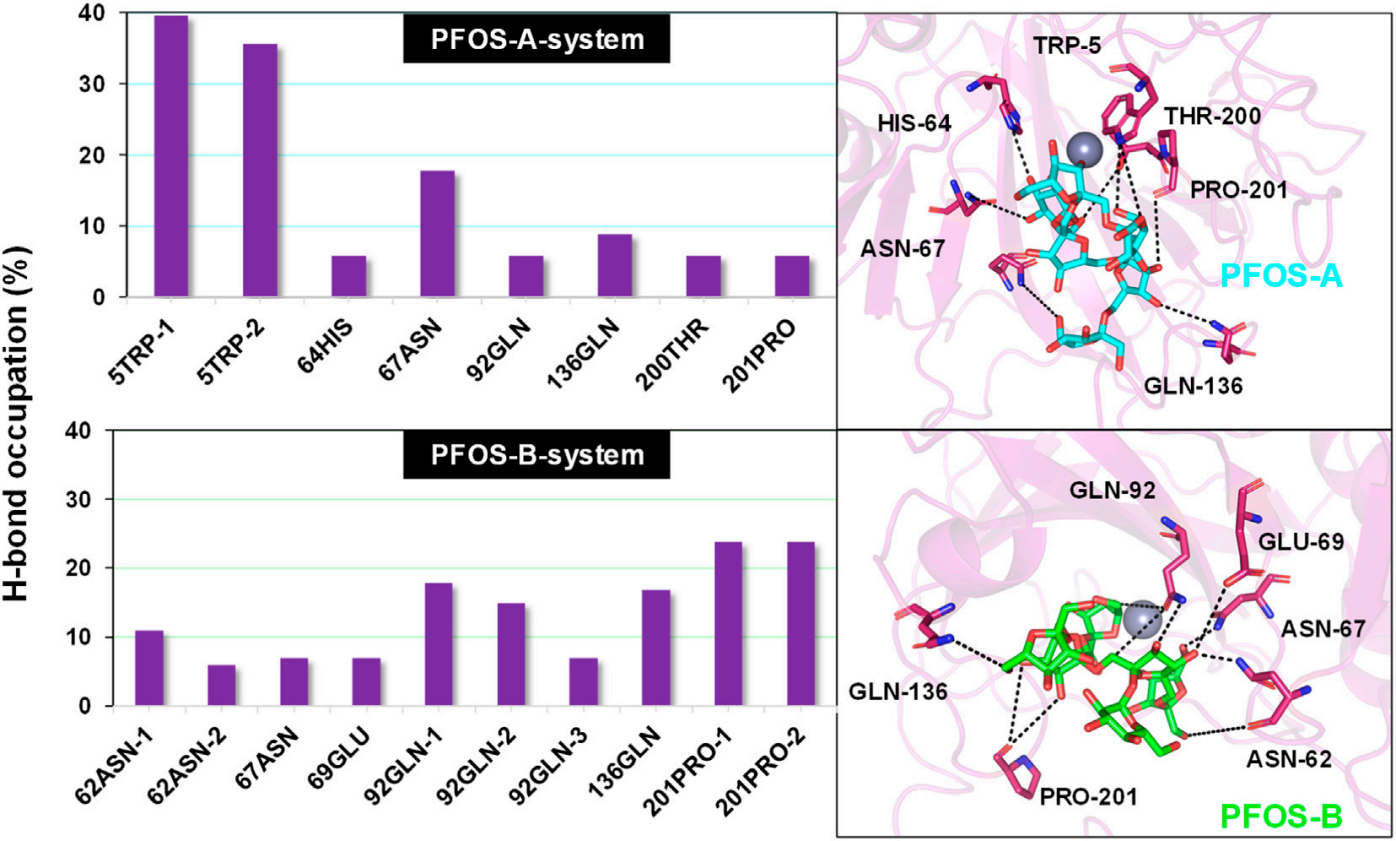
Percentage of intermolecular H-bond occupation of the CA II residues contributing to PFOS-A and PFOS-B. The 3D representative structures illustrating the H-binding between each small molecule and the CA II protein were extracted from the last snapshot of the MD simulation. The black dashed line indicates H-bond formation in the simulation process.

In PFOS-A system, three main hydrogen bonds formed, including H-N2 of ASN-67 with O4 and H-N2 of TRP-5 with adjacent O72 and O70, respectively (Figure 5). This observation is corroborated by the aforementioned energy decomposition analysis, which revealed the negative electrostatic energy contributions from these residues, particularly notable for 5TRP with a value of −1.94 kcal/mol. Conversely, in the PFOS-B and CA II complex, the formation of hydrogen bonds was chiefly facilitated by PRO-201, GLN-92, and GLN-136. Notably, more hydrogen bonds are present in system PFOS-B compared to PFOS-A, suggesting that PFOS-B has a stronger electrostatic interaction, in agreement with free energy calculations. However, the most stable H-bond was detected in the PFOS-A system, which persisted for 39.6% of the simulation time. While, the majority of the other hydrogen bonds endured for less than 20% of the simulation duration. In addition, the overall analysis of the H-bonds occupation data in the table indicates general consistency with the results of molecular docking, which also shows the H-bonds formation of HIS64, ASN67, THR200, and PRO201 in PFOS-A system and ASN62, ASN67, GLN92 and GLN 136 in PFOS-B system.

Considering the presence of multiple hydroxyl groups in PFOS, the formation of hydrogen bond largely depends on the structure and conformation of PFOS, which may be dynamic. Whereas, the ASN67, GLN92, GLN135 and PRO201 showed as the most potential residue to form hydrogen bond with PFOS.

### 3.7 Inhibition mechanism

Based on the above analysis, it can be concluded that the van der Waals interaction domain governs the binding of PFOS molecules, while the electrostatic energy contribution resulting from the formation of hydrogen bonds also has a crucial influence on the binding of PFOS molecules to the hydrophilic residue.

**FIGURE 6:**
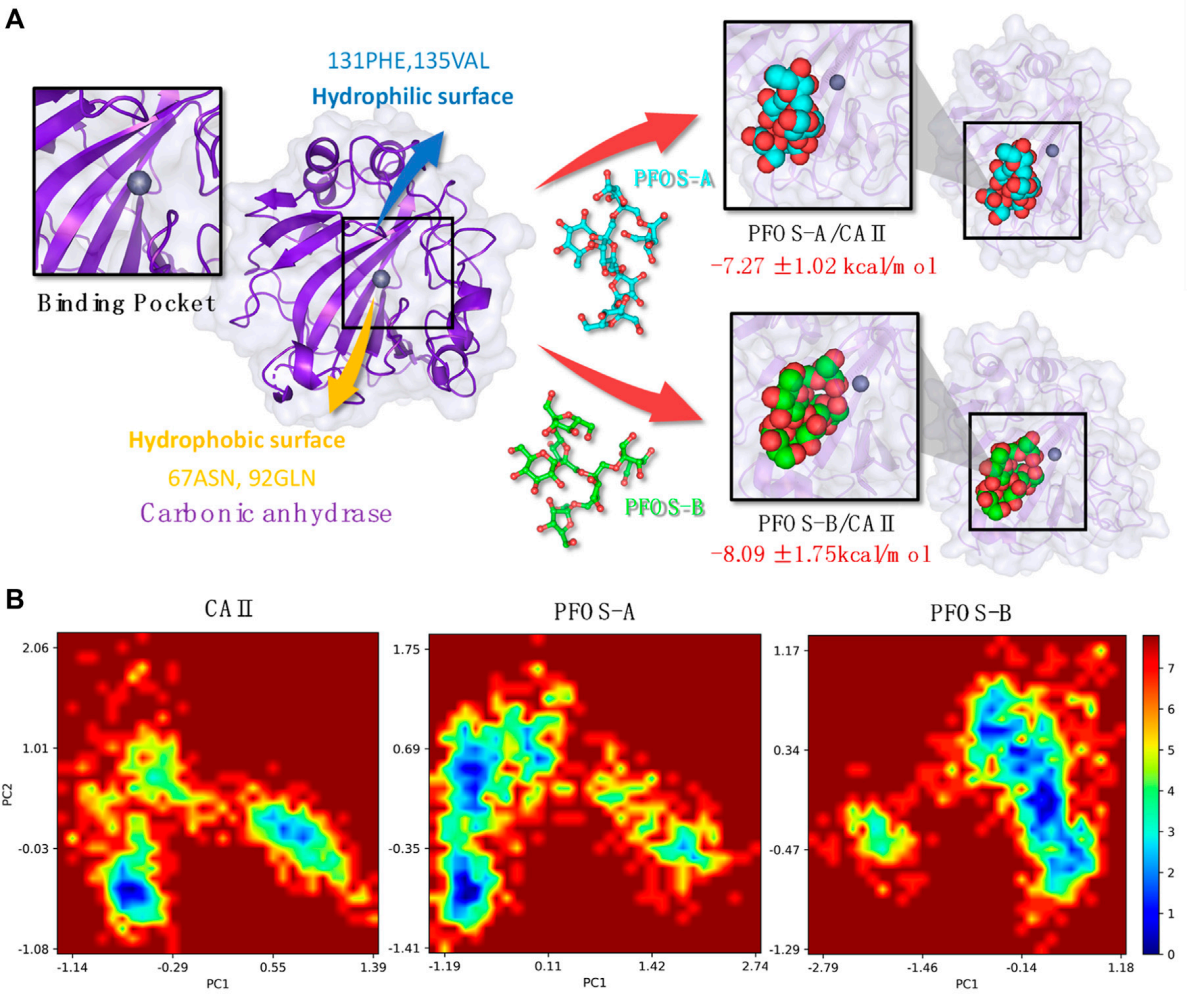
**(A)** Plot of the CA II and PFOS/CA II complexes [right: PFOS-A system (top panel) and PFOS-B system (bottom panel)] demonstrating the interaction mechanism in the binding pocket. **(B)** The free energy landscape of CA II, PFOS-A, and PFOS-B systems. PC1 and PC2 represent principal components 1 and 2, respectively.

By integrating the comprehensive analyses of both van der Waals and electrostatic interactions, the elucidation of the binding site and interaction mechanism between PFOS small molecules and the CA II protein has been elucidated in Figure 6A. Both PFOS structures effectively bind to the entrance of the pocket that accommodates the Zn ion within CA II. While, the double-branched fusiform PFOS-B structure is better at blocking the pocket. The small molecules bind to the hydrophobic surface of the protein through van der Waals interactions and form hydrogen bonds with the hydrophilic surface through electrostatic interactions. This dual interaction strategy effectively contributes to the binding of PFOS, which blocks the carbon dioxide conversion pathway and then results in the inhibition of CA II activity.

Based on the characterization of the binding pocket, the free energy landscapes (FEL) of the three systems, CA II, PFOS-A, and PFOS-B, were analyzed to further investigate the impact of the small molecules on the overall conformational dynamics of the CA II protein and also furnish a qualitative description of their inhibitory potential. The FEL was constructed by projecting the 1000 snapshots from the MD simulation onto the first two principal components, which were employed as the coordinates, as illustrated in Figure 6B.

In the FEL of the PFOS-B system, a distinct blue region indicates the high probability of the existence of a stable conformation. In contrast, the two prominent conformational regions were identified for CA II in the absence of an inhibitor molecule, indicating the stabilizing effect of PFOS-B binding. In the case of the PFOS-A system, its conformational space is characterized by a large region containing two distinct energy traps. This means that the PFOS-A system has a comparatively lower stability. In addition, mirroring the results of the RMSF analysis, the FEL of the PFOS-A system shows numerous smaller basins that are absent from the FEL graph of the PFOS-B system, implying a higher flexibility of the complex binding with PFOS-A.

## 4 Conclusion

In this work, the inhibition effect of PFOS on CA II protein activity was investigated through semi-flexible molecular docking, MD simulations, and MM-PBSA methods. Although the biological activity of PFOS is relatively weaker compared to typical CA II inhibitors, both structures of PFOS bind to the active pocket of CA II with high affinity (PFOS-A: −7.27 ± 1.02 kcal/mol, PFOS-B: −8.09 ± 1.75 kcal/mol). The RMSD and RMSF analysis affirmed the stabilizing effect of PFOS on the CA II structure. These results confirm the spontaneity of the binding process and the stability of the combination in the solvent, indicating the inhibitory potential of PFOS molecules.

The analysis of the binding modes revealed the importance of the three key components of CAII: the C-, D-, and G-sheets, on which a dual interaction strategy was identified by energy decomposition and hydrogen bond occupation analysis. In the binding process between PFOS and CAII, van der Waals interactions form on the hydrophobic surface of CAII mainly with 131PHE and 135VAL, while hydrogen bonds form on the hydrophilic surface mainly with 67ASN, 92GLN, 136GLN and 201PRO. This dual interaction strategy can effectively contribute to the binding of PFOS, resulting in the block of the channel where the CO_2_ conversion reaction takes place and thus inhibiting the activity of CA II. Finally, the FELs of the three systems were analyzed to further determine the effect of the PFOS on the overall conformational dynamics of CA II proteins, demonstrating that both PFOS structures were effective in stabilizing CA II protein into one conformation with low free energy. The inhibitory effect of fructooligosaccharides on the activity of the aging associated protein CA II may provide a mechanistic basis for the anti-aging activity of P. sibiricum.

This study provides novel mechanistic insights into the anti-aging effects of P. sibiricum, paving the way for further research and development of P. sibiricum products. Further elucidation of the pharmacological mechanisms of other small-molecule bioactive compounds in traditional Chinese medicine is also of great importance, which would facilitate the application of traditional Chinese medicine.

## Data Availability

The original contributions presented in the study are included in the article/Supplementary Material, further inquiries can be directed to the corresponding authors.
