# Supplementary material for: Identification of the inhibition mechanism of carbonic anhydrase II by fructooligosaccharides
Source: Front Mol Biosci. 2024 May 28;11:1398603. doi: 10.3389/fmolb.2024.1398603 (PMC11165268; doi:10.3389/fmolb.2024.1398603)
Supplement: Supplementary file 1 [file DataSheet1.PDF]

Supplementary Material

Identification of the inhibition mechanism of carbonic anhy-drase II by fructooligosaccharides

Yue Mu[1], Qingyang Meng[2,*], Xinyi Fan[2], Shuyun Xi[2], Zhongli Xiong[3], Yihua Wang[3], Yanling Huang[3], and Zhen Liu[1,*]

1 School of Chemical Engineering, East China University of Science and Technology, Shanghai, 200237, China

2 Shanghai Pechoin Biotechnology Co., LTD, Shanghai, 200333, China

3 Shanghai Zhengxin Biotechnology Co., LTD, Shanghai, 201612, China

*Correspondence: mengqy@pechoin.com; liuzhen@ecust.edu.cn

1. Preparation of structures

Well-resolved structure of the human carbonic anhydrase II (PDB ID: 12CA) was obtained from the Protein Data Bank (PDB) to be used as initial structures for molecular dynamics (MD) simulations.

PDB DOI: https://doi.org/10.2210/pdb12CA/pdb

The geometries of the fructooligosaccharides in polygonatum sibiricum (PFOS) are optimized at B3LYP/6-31g(d,p) level of theory with the London-dispersion correction by Grimme in Gaussian09 software. The atomic coordinates of the optimized molecules are provided in the end.

2. Validation of the docking method

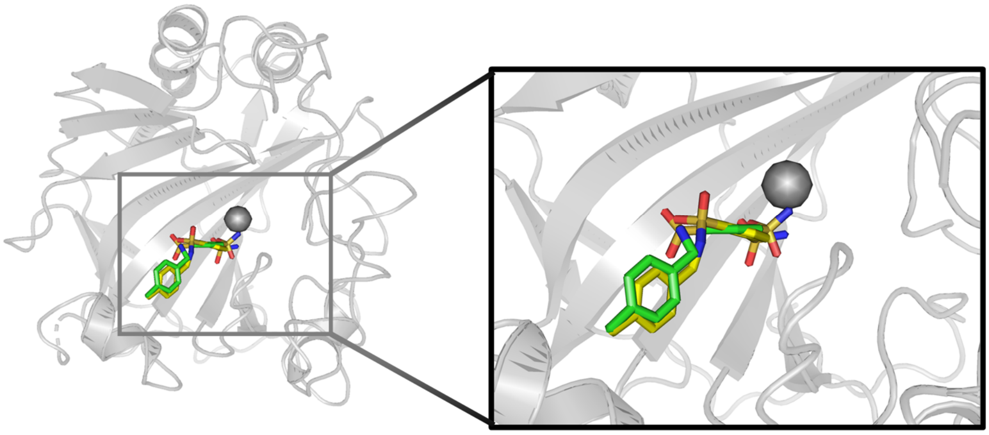

Figure S1. Overlap of the vina-docked pose and native conformation from the crystal structure (PDB ID: 12CA). The docked pose and native confirmation are colored yellow and green, respectively.

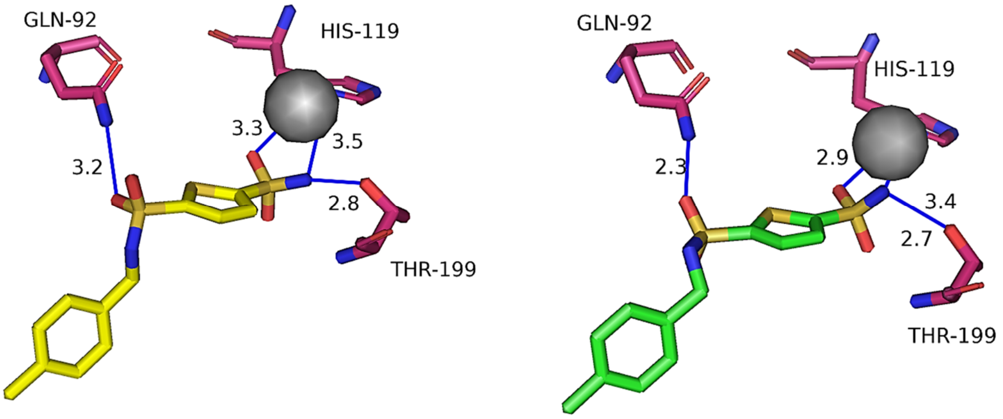

Figure S2. Binding modes and interactions between the inhibitor AL5 and CAII. The docked structure and native structure are colored yellow and green, respectively. The CAII residues interacted with AL5 is highlighted in purple. Hydrogen bonding interactions are visualized as blue lines.

3. Quantum chemical descriptors

Table S1. Quantum chemical descriptors of PFOSs.

Residue	ΔEgap(eV)	Hardness (η)	Softness (S)	Electrophilicity Index (ω)
PFOS-A	0.264	0.132	7.576	0.045
PFOS-B	0.251	0.126	7.961	0.038

The energy gap (ΔE_{gap}) is given by: $\Delta E_{gap} = E_{LUMO} - E_{HOMO}$. The chemical hardness η, chemical softness S and electrophilicity index ω can be evaluated from these HOMO and LUMO energy values, as shown in equation S1-3.

$$\eta = \frac{E_{LUMO} - E_{HOMO}}{2} \quad (1)$$

$$S = \frac{1}{\eta} \quad (2)$$

$$\omega = \frac{\mu^2}{2\eta} \quad (3)$$

4. Energy decomposition

Table S2. PFOS-A system energy decomposition (kcal/mol).

Residue	ΔE_{vdw}	ΔE_{ele}	ΔE_{PB}	ΔG
5TRP	-2.564	-1.944	3.346	-1.162
202PRO	-1.312	-0.585	0.768	-1.130
20PHE	-1.063	-0.205	0.319	-0.948
131PHE	-1.404	-0.005	0.577	-0.832
135VAL	-0.507	-0.009	-0.047	-0.563
198LEU	-0.317	-0.167	0.001	-0.482
64HIS	-0.331	-0.723	0.787	-0.267
132GLY	-0.553	0.135	0.180	-0.238
203LEU	-0.097	-0.126	0.012	-0.211
204LEU	-0.385	-0.103	0.283	-0.205

Table S3. PFOS-B system energy decomposition (kcal/mol).

Residue	ΔE_{vdw}	ΔE_{ele}	ΔE_{PB}	ΔG
131PHE	-2.302	-0.199	0.856	-1.645
135VAL	-1.217	0.143	-0.140	-1.215
198LEU	-1.106	-0.341	0.386	-1.061
202PRO	-0.710	-0.100	0.181	-0.629
204LEU	-0.675	0.099	-0.047	-0.623
7TYR	-2.019	-0.625	2.034	-0.609
200THR	-1.320	-0.382	1.112	-0.591
141LEU	-0.648	-0.085	0.220	-0.512
5TRP	-0.589	-0.459	0.538	-0.510
246ARG	-0.021	-0.707	0.255	-0.472

5. Hydrogen bond occupancies

Table S4. Hydrogen bond occupancies in PFOS-A system.

Donor	Hydrogen	Acceptor	Occupancy%
5TRP@NE1	5TRP@HE1	PFOS-A@O72	39.6
5TRP@NE1	5TRP@HE1	PFOS-A@O70	35.6
67ASN@ND2	67ASN@HD21	PFOS-A@O4	17.8
136GLN@NE2	136GLN@HE21	PFOS-A@O8	8.9
64HIS@NE2	64HIS@HE2	PFOS-A@O3	5.9
92GLN@NE2	92GLN@HE21	PFOS-A@O12	5.9
200THR@OG1	200THR@HG1	PFOS-A@O98	5.9
PFOS-A@O7	PFOS-A@H115	200PRO@O	5.9

Table S5. Hydrogen bond occupancies in PFOS-B system.

Donor	Hydrogen	Acceptor	Occupancy%
PFOS-B@O18	PFOS-B@H11	201PRO@O	23.8
PFOS-B@O19	PFOS-B@H12	201PRO@O	23.8
PFOS-B@O31	PFOS-B@H20	92GLN@OE1	17.8
PFOS-B@O24	PFOS-B@H15	136GLN@OE1	16.8
PFOS-B@O29	PFOS-B@H18	92GLN@OE1	14.9
62ASN@ND2	62ASN@HD21	PFOS-B@O7	10.9
67ASN@ND2	67ASN@HD21	PFOS-B@O4	6.9
92GLN@NE2	928GLN@HE21	PFOS-B@O9	6.9
PFOS-B@O6	PFOS-B@H2	69GLU@OE2	6.9
62ASN@ND2	64ASN@HD21	PFOS-B@O6	5.9

6. Off-target effect

Various human CA isoforms could serve both as potential drug targets and off-targets. Some isoforms, such as CA I and CA II, are ubiquitous, which complicates their targeting. For instance, while inhibiting CA IX and XII is desirable in tumors, it is crucial to avoid inhibiting systemic isoforms like CA I, II, VA, and VB to prevent undesired side effects. Therefore, considering off-target effects is crucial for assessing the potential undesired side effects of inhibitors. To this end, we preliminarily assessed the potential off-target effects on CA I through the simple molecular docking method, which is the most possible off-target of CAII inhibitor according to previous research.

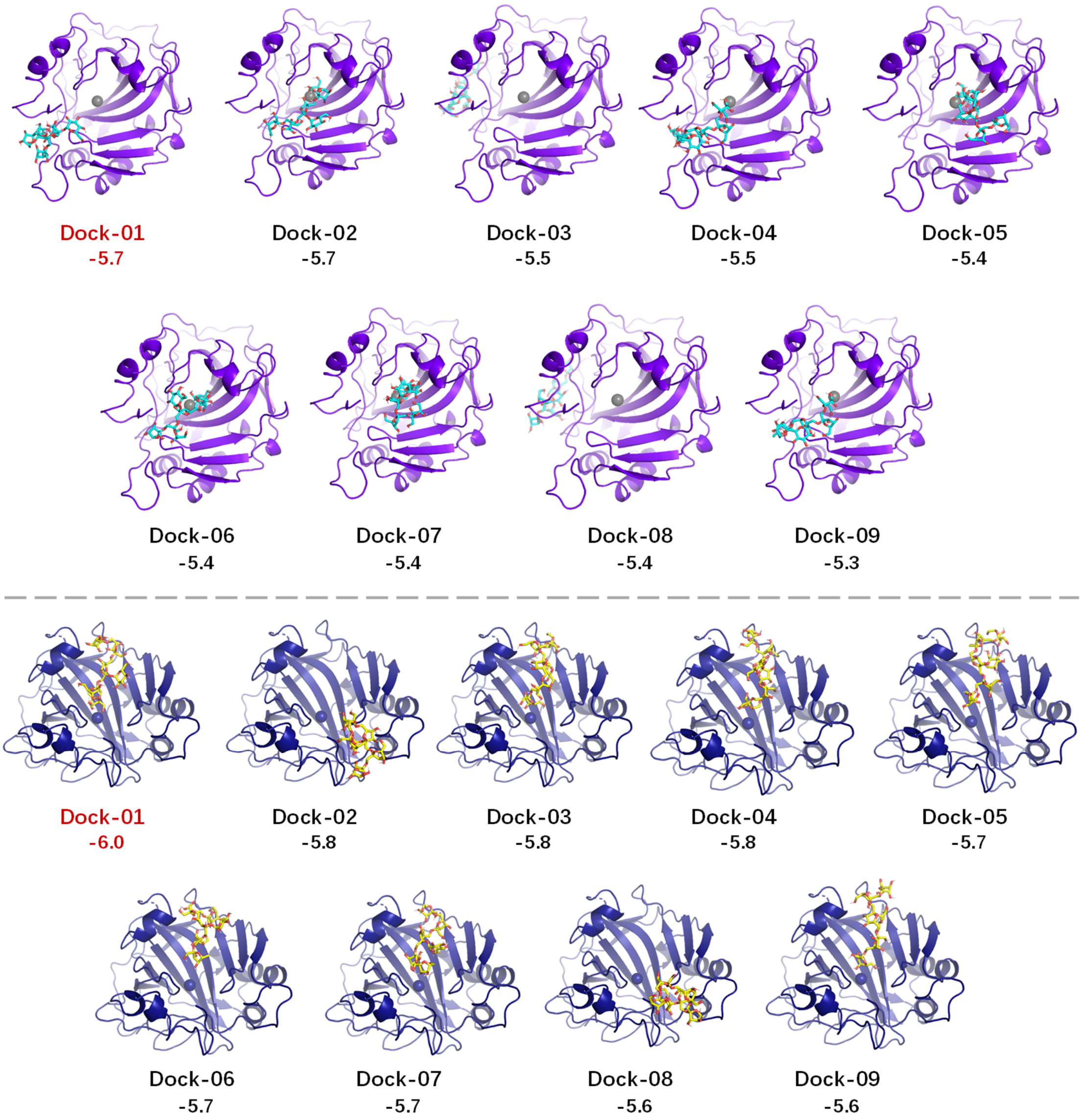

Figure S3: Molecular docking of PFOS with CA I (shown in purple) and CA II (in dark blue). Docking scores are reported in kcal/mol.

The docking scores for both CA I and CA II with PFOS are similar; however, CA I tends to dock predominantly outside the active pocket, minimally impacting its enzymatic activity. This indicates a modest preference for CA II, though more comprehensive studies are necessary to confirm this observation accurately. The lack of marked selectivity in the docking scores aligns with the behavior of current carbonic anhydrase inhibitor, which generally do not show strong preference for specific isozymes. Actually, any potential side effects of natural small molecules PFOS on non-target proteins are likely to be minimal.

Structures of the PFOSs

PFOS-A

129

C	4.92138000	1.66319400	1.53726500
C	5.95674900	1.02928500	0.60761800
C	6.27808900	-0.31082000	1.29756100
C	7.65818600	-0.84233000	0.90349700
O	7.79422200	-0.86628400	-0.50634900
O	0.26132700	-0.18741800	0.30417700
O	0.18507200	3.73626800	0.61248100
O	0.06016100	3.43223900	-3.06370500
O	2.55438500	4.83013800	-2.58946400
O	3.69015800	4.60585800	-0.06804600
O	-1.10640400	0.81314300	-2.87282300
C	5.21417800	1.01318700	2.90002300
O	0.30402500	-1.94453100	-2.83304100
O	3.42722700	-3.42003200	0.34378500
O	5.64652800	-2.04482400	-1.74804000
O	5.64999400	-5.40995500	-0.42450400
O	5.25194000	-1.23648100	0.94252500
O	6.24201900	-0.00065800	2.67169300
C	5.15579200	-2.39859100	1.76051400
C	3.99167000	0.36319300	3.53952600
O	5.49488300	0.94508000	-0.72458300
O	5.05622700	3.07109800	1.65597900
O	4.34738800	-0.40410200	4.67842100
C	4.73039700	-3.60070300	0.93253200
C	5.63044200	-3.99712300	-0.25165500
C	5.00393500	-3.27176600	-1.46469400
C	3.51154400	-3.07449400	-1.02821500
C	2.53876100	-4.00462000	-1.77062800
O	2.44230300	-3.71963800	-3.13692900
O	3.19413700	-1.70920300	-1.28432700
C	2.43990000	-0.94886900	-0.34303600
C	0.94330500	-1.11729800	-0.56853000
C	0.48447700	-0.80176600	-2.00877900
C	-0.79730000	-0.01868700	-1.77304900
C	-0.51995200	0.71237700	-0.44891800
C	-1.77013500	1.00177800	0.39611200
O	-2.80842400	1.59078400	-0.40526200
C	-3.96190600	0.83140700	-0.65944400
C	-4.65575300	1.39169200	-1.93542200
C	-5.21844700	0.14050000	-2.66025100
O	-6.60748600	-0.07714300	-2.48670600
C	-4.43114700	-1.03816300	-2.04277400
C	-3.51536500	-1.70603000	-3.07189000
O	-2.61373100	-2.65441300	-2.50296100
O	-3.62238600	-0.50774400	-0.98541800
O	-3.64834300	2.02639500	-2.71728700
C	-4.86655700	0.82770700	0.57791400
O	-5.86243100	-0.14347700	0.31364500
C	-7.05786200	-0.10310200	1.04703900
C	-7.72494800	-1.52079800	0.96885100
C	-7.90677100	-1.90371500	2.45357000
C	-6.80294500	-1.09763400	3.14174800
C	-5.42744100	-1.76132100	3.08477200
O	-5.51720300	-2.93147700	3.89195000
O	-6.81306400	0.14633300	2.43034500
O	-9.19996500	-1.43850900	2.84517900
O	-6.88027600	-2.43593400	0.28527600
C	-7.98077600	1.01169500	0.54325800
O	-9.29423900	0.90176700	1.04964700
O	0.28119300	1.84701600	-0.75649100
C	-0.19416800	3.17660300	-0.62696200
C	0.41770400	3.95307500	-1.79889600
C	1.93392600	3.99519200	-1.61782700
C	2.27117700	4.56485200	-0.24794300
C	1.60996300	3.72854800	0.85651500
C	1.82748400	4.25940900	2.28926900
O	2.70792900	3.46941200	3.06148200
H	4.05814300	4.91323200	-0.91091000
H	2.22779500	4.52667200	-3.44943100
H	-0.03905000	2.46261000	-2.98256600
H	-1.96249200	1.25538200	-2.70805000
H	7.10979900	-1.46231300	-0.88319000
H	-0.57059600	-2.33495000	-2.65905600
H	4.99724300	-1.35106400	-1.51193800
H	4.73379400	-5.71378900	-0.48670200
H	5.15516800	-0.87816800	4.43210400
H	6.25374000	0.61796000	-1.23386400
H	4.66736300	3.50139100	0.86455500
H	1.80651300	-2.98384900	-3.21520200
H	-7.08422500	0.55152100	-3.04423200
H	-3.11218600	-3.28118600	-1.96229300
H	-4.03587200	2.34068400	-3.54414500
H	-4.67527500	-3.39997300	3.84117600
H	-9.29900200	-1.58733200	3.79503500
H	-7.41816300	-3.21227500	0.08468800
H	-9.24007700	0.57381100	1.96184700
H	3.60745000	3.52926900	2.68341000
H	3.91737400	1.39202300	1.18429100
H	6.86487800	1.64480500	0.67615000
H	7.83840600	-1.82931400	1.35522100
H	8.41576300	-0.16050400	1.29907900
H	5.65508500	1.74645400	3.58046000
H	4.41843200	-2.23245900	2.55512000
H	6.11011600	-2.63467000	2.24698100
H	3.48390500	-0.25524500	2.78256000
H	3.29329000	1.14707000	3.84842700
H	4.65246700	-4.45135200	1.62029200
H	6.67216600	-3.69539400	-0.11988900
H	5.06436400	-3.91313100	-2.34867100
H	1.56824800	-3.96209200	-1.25486000
H	2.91496000	-5.03088600	-1.66016900
H	2.71273800	0.09359000	-0.52659900
H	2.70963000	-1.20143400	0.68230000
H	0.62323400	-2.13170400	-0.30048700
H	1.22363400	-0.17305900	-2.51314000
H	-1.59509800	-0.73408300	-1.57757700
H	-1.52082200	1.69758100	1.20194700
H	-2.08700500	0.04934100	0.82230200
H	-5.45977100	2.09302800	-1.68055600
H	-4.98087000	0.23574100	-3.73173700
H	-5.15178300	-1.74197500	-1.60907000
H	-2.87753500	-0.95212500	-3.54327500
H	-4.14089000	-2.16875500	-3.85067700
H	-5.26997800	1.83192700	0.74825300
H	-4.27552400	0.54866300	1.45771400
H	-8.70598000	-1.43357500	0.49165200
H	-7.79507300	-2.98290300	2.60938900
H	-7.04928900	-0.88050300	4.18720000
H	-4.68649900	-1.04567200	3.47229700
H	-5.17606500	-1.99164000	2.04155700
H	-8.02638800	0.92719000	-0.54612400
H	-7.52399300	1.98019200	0.80086500
H	-1.28345700	3.22413700	-0.66499900
H	0.04131000	4.98221500	-1.75445100
H	2.33107700	2.97076100	-1.68134400
H	1.87188900	5.58880500	-0.18615500
H	1.97540500	2.69510500	0.81688300
H	0.85446100	4.22985400	2.78838700
H	2.15029400	5.31151400	2.25201300

PFOS-B

129

O	1.17517700	0.44442800	1.21660300
O	2.30707500	4.01754800	0.81474800

O	1.74267000	3.34589000	-2.77137300
O	4.59785500	3.88863100	-2.62262200
O	5.80822900	3.40695100	-0.12525700
O	0.20800900	0.77668900	-2.24480300
O	1.56555500	-1.94193100	-1.42469100
O	5.53127500	-1.68364100	1.99236600
O	6.27370300	-1.73204800	-1.36276500
O	7.95024800	-3.28123400	1.43788600
O	6.47951500	0.62961600	0.15027500
C	6.88818400	0.35001900	1.48356000
C	6.86077300	-1.14486300	1.80401000
C	7.45645400	-2.11789000	0.77554600
C	6.25951500	-2.44865600	-0.16193400
C	5.01511700	-2.15167000	0.75492600
C	4.11377800	-3.34610900	1.08463800
O	3.41554700	-3.83748400	-0.04046800
O	4.24584400	-1.16883500	0.08810600
C	3.44239600	-0.30605100	0.87890200
C	1.97439600	-0.60456400	0.62265200
C	1.63536500	-0.63572300	-0.88715600
C	0.36106500	0.21310600	-0.95721900
C	0.62887900	1.22541500	0.18238800
C	-0.57814000	1.92944400	0.79448100
O	-1.29132500	2.67146000	-0.19909100
C	-2.58482900	2.23982000	-0.53933900
C	-2.91048400	2.73420400	-1.97126400
C	-3.81882100	1.62672100	-2.56211700
O	-5.18754900	1.94323700	-2.57928700
C	-3.49355900	0.38578100	-1.69004800
C	-2.83622500	-0.78883000	-2.38536400
O	-2.92431000	-1.87780900	-1.45182900
O	-2.62812300	0.82354800	-0.62824700
O	-1.66668400	2.79986600	-2.66466400
C	-3.54896500	2.72500900	0.55253900
O	-4.83902600	2.18434000	0.29266600
C	-5.67533000	1.93385900	1.41204100
C	-6.83321900	0.99110500	0.94553800
C	-6.75623600	-0.19252400	1.93170300
C	-5.30154100	-0.16416100	2.40327600
C	-4.33651400	-0.91644100	1.49074400
O	-4.68727500	-2.29153600	1.50273000
O	-4.99296400	1.24237000	2.44871500
O	-7.65305800	0.11603900	3.00146900
O	-6.61609400	0.59165600	-0.40189700
C	-6.17708100	3.24898900	2.01805100
O	-7.24749600	3.05312100	2.91670300
O	1.63676600	2.08736600	-0.34090800
C	1.63746900	3.49710500	-0.32057300
C	2.36680200	3.87916000	-1.61932900
C	3.82405700	3.41288100	-1.52899400
C	4.47164900	3.93325100	-0.25755100
C	3.64870400	3.53401000	0.97306900
C	4.20125700	4.09698700	2.28145900
O	5.41495200	3.45685600	2.64916700
H	6.25037200	3.58098900	-0.97145600
H	4.11203800	3.63873500	-3.42267600
H	1.47813600	2.42754100	-2.56502500
H	-0.50893800	1.44229500	-2.23354800
H	0.65518300	-2.28417500	-1.35267900
H	6.15172200	-0.79770900	-1.11547900
H	7.23816700	-3.59787700	2.01199600
H	3.05749900	-3.08496900	-0.55122700
H	-5.53217600	1.85263500	-1.67254000
H	-1.83160000	3.03843300	-3.58589800
H	-4.57522100	-2.64333600	0.60101800
H	-7.57086900	-0.57745800	3.66963100
H	-7.43420300	0.20417000	-0.73679600
H	-7.07811700	2.23867600	3.41670900
H	6.03383500	3.58564000	1.91492100
H	6.14735900	1.54352300	0.10399500
C	-2.23779000	-3.06825200	-1.67432400
C	-3.00602200	-4.22515500	-0.96782400
C	-0.81520700	-4.16402600	-0.12812600
C	-2.24424600	-4.44043900	0.33064500
H	-2.88684200	-5.14201700	-1.55960700
H	-0.45107700	-5.02085600	-0.71196500
H	-2.55732000	-3.67221600	1.05508000
O	-0.96160700	-3.01826300	-0.98627100
O	-4.38680600	-3.92109200	-0.81388400
H	-4.77224900	-4.68218400	-0.35429200
O	-2.46535900	-5.74630300	0.81986500
H	-1.82540100	-5.86396100	1.54059800
C	0.15069700	-3.86234300	1.00718600
H	1.15542700	-3.66858100	0.62300000
H	-0.20290100	-2.98527000	1.56037200
C	-1.94501600	-3.35350600	-3.15752100
H	-2.88398600	-3.31293000	-3.72548600
H	-1.27567400	-2.57504100	-3.53377400
O	-1.26857600	-4.59070300	-3.31691100
H	-1.91453400	-5.27236400	-3.53494500
O	0.15236000	-4.97309700	1.91596900
H	0.84011100	-5.58305300	1.61818200
H	6.24820600	0.87341100	2.20648300
H	7.92393100	0.69204300	1.64670200
H	7.37280500	-1.26758400	2.76458600
H	8.28516600	-1.70624900	0.19705000
H	6.29688300	-3.51037700	-0.41916900
H	3.43226000	-3.03086300	1.89376900
H	4.72502900	-4.16683300	1.47672300
H	3.65099200	0.70975400	0.54124200
H	3.68794500	-0.37181500	1.94094600
H	1.68151800	-1.54816700	1.08861200
H	2.41773200	-0.11741000	-1.44749900
H	-0.50817400	-0.39196800	-0.67350300
H	-0.23412300	2.62269100	1.56859700
H	-1.19835100	1.15964800	1.25613200
H	-3.42476700	3.70137900	-1.97610600
H	-3.52708400	1.46884800	-3.60787600
H	-4.43768900	0.03359200	-1.26739000
H	-1.80001900	-0.54523400	-2.64280000
H	-3.39740600	-1.02268000	-3.29997800
H	-3.56655700	3.82180700	0.56204800
H	-3.17813800	2.36281800	1.51463100
H	-7.79277100	1.50249800	1.06781500
H	-7.00871000	-1.14208600	1.44621400
H	-5.21152000	-0.54341400	3.42759200
H	-3.31045200	-0.75406000	1.85010600
H	-4.39505900	-0.48957800	0.49346000
H	-6.53812800	3.88869300	1.20631100
H	-5.31953900	3.74837900	2.49579400
H	0.62800200	3.90902800	-0.29990300
H	2.35509900	4.97016200	-1.72063700
H	3.83487900	2.31265000	-1.49787800
H	4.52170700	5.03090100	-0.31018400
H	3.64347000	2.44508200	1.06470700
H	3.47600600	3.89741000	3.07499000
H	4.31588800	5.18980600	2.19987400
